# OA20 Combining B cell depletion and JAKi for difficult-to-treat rheumatoid arthritis

**DOI:** 10.1093/rap/rkad070.020

**Published:** 2023-09-27

**Authors:** Sook Yan Lee, Sarah Shakour, Amelia Holloway, Arti Mahto

**Affiliations:** Kings College Hospital NHS Foundation Trust, London, United Kingdom; Maidstone and Tunbridge Wells NHS Trust, Maidstone, United Kingdom; Kings College Hospital NHS Foundation Trust, London, United Kingdom; Kings College Hospital NHS Foundation Trust, London, United Kingdom

## Abstract

**Introduction:**

Difficult-to-treat rheumatoid arthritis (D2T RA) remains an area of unmet need in rheumatology. Despite the increasing availability of biologic and targeted synthetic disease-modifying anti-rheumatic drugs (DMARDs) choices to treat RA, a significant proportion of patients with D2T RA still fail to achieve low disease activity or remission [1]. Here we present the case of a patient with D2T RA refractory to multiple lines of conventional DMARDs (cDMARDs) and biological DMARDs (bDMARDs), who has been managed with a novel combination of rituximab and filgotinib, with no adverse side effects or increased infection risk frequency in his follow up period to date.

**Case description:**

A 51-year-old man was referred with a history of strongly seropositive RA which had failed multiple conventional treatments. He was diagnosed with seropositive RA in his teens and by the age of 23, had extensive deforming arthritis with fixed flexion in his knees for which he has had required arthroplasties. Previous cDMARDs include methotrexate, leflunomide, sulphasalazine and hydroxychloroquine, all of which were stopped due to side effects or inefficacy. He had primary failure to etanercept, adalimumab, tocilizumab, abatacept, and rituximab. He previously had good efficacy on the combination of infliximab and methotrexate but developed secondary failure after a decade. Golimumab and methotrexate worked well in combination but was stopped when methotrexate was discontinued due to a persistent macroscopic haematuria. Other comorbidities include type 1 diabetes, hypercholesterolaemia, hypothyroidism, and osteoporosis.

On initial review he was on a combination of baracitinib and sulphasalazine. However, he had evidence of persistent high disease activity with a disease activity score (DAS-28) of 6.09 with 9 tender joints, 4 swollen joints, and VAS 7/10. Inflammatory markers were elevated with an ESR of 60 mm/hr and CRP of 16mg/L.

He switched to upadacitinib, but this was discontinued due to headaches and lack of clinical benefit. He was re-trialled on infliximab with concurrent tacrolimus, but this was not effective and he had anti-infliximab antibodies of > 200ng/mL (0-10 ng/mL) on immunological testing. Combination rituximab (1 gram days 1 and 15) and cyclophosphamide (750mg days 3 and 17) was initiated next, which helped with his jaw synovitis, but not the remainder of his joints, and developed gastrointestinal side effects with the cyclophosphamide. Following MDT discussion, filgotinib was introduced and he has managed to reduce his prednisolone dose by 3mg to his current 5mg dose, with no significant deterioration in his joints and an improvement in his inflammatory markers.

**Discussion:**

Our patient fulfils the EULAR definition of D2T RA with failure to multiple biologic and conventional DMARDs, persisting clinical and biochemical evidence of active disease and difficulties with tapering glucocorticoid treatment [2]. This has had a significant impact on his quality of life and activities of daily living. Insulin dependent diabetes adds to the complexities of his management, as though his diabetes is well controlled at baseline with the use of a closed loop insulin system, the recurrent need for steroids had a knock-on sequela on his blood glucose control, and further contributed to his osteoporosis and cardiovascular risk.

B cells have been identified as key contributors in the development of RA [3]. The synovial membrane of RA patients is known to contain B cell derived plasma cells which produce RF, with RF positivity being associated with aggressive articular disease and increased extra-articular manifestations [4]. Following the promising outcomes demonstrated in a randomized, double blind, controlled study by Edwards et al [5], we attempted B cell depletion with rituximab in combination with cyclophosphamide. Unfortunately, the cyclophosphamide could not be continued as he developed gastrointestinal side effects and the combination only conferred marginal benefit. Blood tests done 6 months following rituximab did show a depletion in his B cell count from 294 to 0, and in his RF from 864 to 276.

Given the infection risks with prolonged depletion of his peripheral blood B cell counts, filgotinib was introduced given its rapid onset of action and short half-life, with the benefits of JAKi over other classes of DMARDs in D2T RA patients being demonstrated by Ochi et al [6]. Reassuringly, there has been no adverse side effects or major infections in his follow up period to date.

**Key learning points:**

This case highlights the complexities of managing a patient with D2T RA. Biologics are now the standard of care in RA, but despite the wide range available, 15% of patients are refractory to treatment, and sustained remission is only achieved in less than 50% [7]. The use of concomitant dual biologic therapy to neutralize multiple inflammatory pathways appears to be a promising strategy, but there is a paucity of evidence on the effectiveness and safety of combining two bDMARDs.

Previous studies have looked at combining rituximab with a TNFi [8], and a TNFi with abatacept or anakinra, but this was associated with more side effects and no clear advantage [9]. Another study has also shown benefit in adding an IL17 inhibitor to RA patients who have not responded to certolizumab [10]. Ours is the first case that we know of, where rituximab has been used in combination with a JAK inhibitor. We are hoping to approach his treatment strategy by depleting his B cells and combine this with targeting the JAK-STAT pathway to reduce any pro-inflammatory cytokine signaling. The short half-life of filgotinib would be an advantage should there be any adverse side effects or severe infections.

Our patient has been strongly engaged in the journey of his RA treatment, but unfortunately, the extent of his previous drug treatments and comorbidities means he is usually excluded from entering any drug trials, and this would similarly be applied to most patients who fall into the D2T RA category. A patient centred treatment approach is especially crucial when undertaking riskier treatment strategies, with all treatment consultations requiring a careful discussion of the risks and benefits. This case emphasizes the need for further research on combination biologic therapies in D2T RA and would add to the growing body of evidence in this area.

